# Comparative Effectiveness of Fidaxomicin vs Vancomycin in Populations With Immunocompromising Conditions for the Treatment of *Clostridioides difficile* Infection: A Single-Center Study

**DOI:** 10.1093/ofid/ofad622

**Published:** 2023-12-08

**Authors:** Majd Alsoubani, Jennifer K Chow, Angie Mae Rodday, David Kent, David R Snydman

**Affiliations:** Division of Geographic Medicine and Infectious Diseases, Department of Medicine, Tufts Medical Center, Boston, Massachusetts, USA; Division of Geographic Medicine and Infectious Diseases, Department of Medicine, Tufts Medical Center, Boston, Massachusetts, USA; Tufts Clinical and Translational Science Institute, Tufts Medical Center, Boston, Massachusetts, USA; Predictive Analytics and Comparative Effectiveness Center, Tufts Medical Center, School of Medicine, Tufts University, Boston, Massachusetts, USA; Division of Geographic Medicine and Infectious Diseases, Department of Medicine, Tufts Medical Center, Boston, Massachusetts, USA; The Stuart B. Levy Center for the Integrated Management of Antimicrobial Resistance, School of Medicine, Tufts University, Boston, Massachusetts, USA

**Keywords:** *Clostridioides difficile*, fidaxomicin, immunocompromised, transplant

## Abstract

**Background:**

*Clostridioides difficile* infection (CDI) is a leading cause of morbidity in immunocompromised hosts with increased risk of complications and recurrences. In this study, we examined the clinical effectiveness of fidaxomicin vs vancomycin in treating CDI in this patient population.

**Methods:**

This single-center retrospective study evaluated patients with CDI between 2011 and 2021. The primary outcome was a composite of clinical failure, relapse at 30 days, or CDI-related death. A multivariable cause-specific Cox proportional hazards model was used to test the relationship between treatment and the composite outcome, adjusting for confounders and treating death from other causes as a competing risk.

**Results:**

This study analyzed 238 patients who were immunocompromised and treated for CDI with oral fidaxomicin (n = 38) or vancomycin (n = 200). There were 42 composite outcomes: 4 (10.5%) in the fidaxomicin arm and 38 (19.0%) in the vancomycin arm. After adjustment for sex, number of antecedent antibiotics, CDI severity and type of immunosuppression, fidaxomicin use significantly decreased the risk of the composite outcome as compared with vancomycin (10.5% vs 19.0%; hazard ratio, 0.28; 95% CI, .08–.93). Furthermore, fidaxomicin was associated with 70% reduction in the combined risk of 30- and 90-day relapse following adjustment (hazard ratio, 0.27; 95% CI, .08–.91).

**Conclusions:**

The findings of this study suggest that the use of fidaxomicin for treatment of CDI reduces poor outcomes in patients who are immunocompromised.

## Teaser

This study evaluated the clinical effectiveness of fidaxomicin vs vancomycin for *Clostridioides difficile* infection in immunocompromised hosts. Our findings confirm that the use of fidaxomicin was associated with a reduced risk of treatment failure and recurrence.

## Graphical Abstract

**Graphical abstract:**
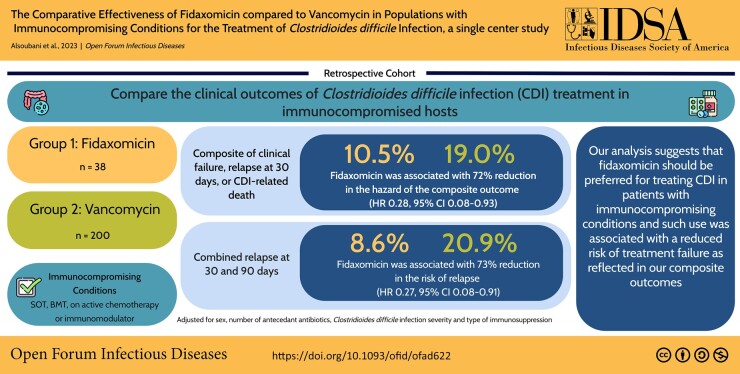
This graphical abstract is also available at Tidbit:https://tidbitapp.io/tidbits/the-comparative-effectiveness-of-fidaxomicin-compared-to-vancomycin-in-populations-with-immunocompromising-conditions-for-the-treatment-of-clostridioides-difficile-infection-a-single-center-study

*Clostridioides difficile* is a leading cause of health care–associated diarrhea worldwide. The Centers for Disease Control and Prevention currently lists it as one of the top pathogens associated with urgent threat due to increased antimicrobial resistance [1]. Patients with immunocompromising conditions are at increased risk of *C difficile* infection (CDI) as compared with other hospitalized patients [2, 3]. For example, the prevalence of CDI in recipients of solid organ transplant (SOT) is up to 7-fold higher when compared with the general population [4, 5]. Furthermore, the prevalence of CDI is up to 20% among recipients of hematopoietic stem cell transplant, as opposed to 1% in the general population [6, 7]. The increased prevalence in this population is likely related to hospitalization, increased exposure to broad-spectrum antimicrobials, and intense immunosuppression in the posttransplant period [8, 9].

Immunocompromised status confers more than twice the risk of recurrence as compared with the general population [3]. The presence of CDI in SOT recipients is associated with worse outcomes, including increased in-hospital mortality, organ failure or rejection, and longer hospital length of stay vs SOT recipients without CDI [10, 11]. Moreover, in recipients of hematopoietic stem cell transplant, there is a strong correlation between CDI and subsequent acute graft-vs-host disease [8]. The risk of complications is increased with each recurrent CDI episode.

An update of CDI treatment guidelines was published in 2021 recommending the use of fidaxomicin over vancomycin for initial and recurrent episodes of CDI for all patients, however this recommendation was conditional with a low certainty of evidence [12]. The recommendations were based on the results of randomized controlled trials that mostly included patients who were immunocompetent without history of transplantation or use of immunosuppressants [13–16]. Evidence on the efficacy of fidaxomicin in populations with immunocompromising conditions is sparse. A post hoc analysis of 2 double-blinded randomized controlled trials testing fidaxomicin against vancomycin for the treatment of CDI in 183 patients with cancer included only 33 who were undergoing active chemotherapy treatment [17]. An observational retrospective study comparing vancomycin and fidaxomicin in 96 recipients of hematopoietic stem cell transplant found no differences in initial or global cure of CDI [18].

Since there are limited data on fidaxomicin effectiveness in patients with immunocompromising conditions, we chose to compare the clinical outcomes of CDI following treatment with fidaxomicin vs vancomycin in patients with immunocompromising conditions.

## METHODS

### Study Setting

This retrospective study was conducted at Tufts Medical Center, a tertiary care academic hospital in Boston, Massachusetts. The study examined patients with immunocompromising conditions who were diagnosed with CDI from 1 March 2011 to 31 December 2021.

Patients were classified as having an immunocompromising condition if they met at least 1 of the following criteria at the time of CDI diagnosis: (1) having a solid organ or hematologic stem cell transplant at any time prior to being diagnosed with CDI; (2) undergoing active chemotherapy for leukemia, lymphoma, or solid tumors; or (3) taking immunomodulatory agents. Patients were excluded if they were <18 years old; did not receive any treatment for CDI; and were treated with metronidazole only, fecal microbiota transplant, or bezlotoxumab.

The Tufts Medical Center Institutional Review Board approved this study (SUDY00001199).

### Definitions

*CDI* was defined as a diarrheal illness with a positive stool assay for *C difficile* that was associated with the initiation of treatment by the treating provider. We reviewed physician notes to confirm that the presence of diarrhea, abdominal pain, or ileus on clinical presentation was consistent with CDI for each case. A test result was considered positive if glutamate dehydrogenase antigen and toxin assays were positive or if a nucleic acid amplification test (NAAT) result was positive [19]. Indeterminate results, characterized by positive glutamate dehydrogenase antigen test result and negative toxin test result, were reflexed to a NAAT. During the entire study period, we used antigen/toxin testing as the first step, followed by NAAT for indeterminate results for the diagnosis of CDI. In August 2020, NAAT reflex for indeterminate results required approval by the antimicrobial stewardship team or infectious disease physician [20].

*Index CDI* was defined as the first episode of CDI meeting our case definition in our study window after the diagnosis of the immunocompromising condition. Patients with previous episodes of CDI, as reported in the clinical notes, were defined as having a history of CDI.

*Clinical failure* was defined as any conversion or additional use of antimicrobials >72 hours after initiation of therapy by the treating physician for perceived treatment failure. *Relapse at 30 days* was defined as recurrence of diarrhea and/or the need to restart CDI treatment within 30 days of stopping therapy for the index CDI case, as determined by the treating physician with or without a positive test result. *Relapse at 90 days* was defined as recurrence of diarrhea and/or the need to restart CDI treatment between 30 and 90 days of stopping therapy for the index CDI case, as determined by the treating physician with or without a positive test result, excluding relapses that occurred before 30 days. *Total relapse* was defined as recurrence of diarrhea and/or the need to restart CDI treatment within 90 days of stopping therapy for the index CDI case, as determined by the treating physician with or without a positive test result.

*Death related to CDI* was defined as any death that was attributed to CDI within 30 days of initial diagnosis. This included death from fulminant colitis or septic shock. *Death from other causes*, a competing risk to the primary outcome, was defined as any death that was not associated with CDI within 30 days of CDI diagnosis. This included cardiac-, pulmonary-, or cancer-related deaths.

*Severe CDI* was defined per the criteria of the Infectious Diseases Society of America (IDSA): leukocytosis with white cell count ≥15 000 cells/mL or serum creatinine >1.5 mg/dL [19]. *Hospital-acquired CDI* was defined as infection diagnosed after 48 hours following admission to the hospital. *Health care–associated CDI* was defined as exposure to a health care facility within 30 days prior to diagnosis.

*CDI treatment* was classified as fidaxomicin or vancomycin if patients received at least 72 hours of the agent.

### Data Collection

CDI cases in patients who were immunocompromised and satisfied our inclusion criteria were abstracted from the microbiology and hospital databases. All demographic and clinical data were collected retrospectively from the electronic medical record: age, sex, race, ethnicity, comorbid conditions, history of CDI, use of gastric acid suppression, toxin test or NAAT positivity for the toxin gene, location prior to and at the time of CDI diagnosis, and intensive care unit admission. The Charlson Comorbidity Index was used to evaluate patients' comorbidities [21]. Antecedent antibiotic exposure was limited to 30 days prior to index case diagnosis. Laboratory data, including white blood cell count and serum creatinine, were collected at the time of CDI diagnosis. If there were multiple values on the same day, the one closest to the time of CDI testing was recorded. Probiotic use was not collected for this study. Study data were collected with REDCap (Research Electronic Data Capture) hosted by Tufts Medical Center. REDCap is a secure web-based application designed to support data capture for research studies [22].

Cases where death occurred in the first 30 days following the diagnosis of CDI were reviewed independently by 2 physicians blinded to treatment (M. A., C. T.). Death was adjudicated as death attributed or contributed to CDI or as death from another cause. There were no discrepancies in adjudication between the reviewers.

### Primary Study Exposure and Clinical Outcomes

The main exposure variable was the use of fidaxomicin or vancomycin for CDI treatment. At Tufts Medical Center during the study period, fidaxomicin was the recommended regimen for patients with increased risk of recurrence, including those who had chronic kidney disease, had a history of CDI, were older, or were immunocompromised. Vancomycin was recommended for all other patients. Physicians were allowed to choose either treatment regimen.

The primary study outcome was a composite outcome of clinical failure, relapse within 30 days following completion of initial CDI treatment, or death due to CDI. Each component of the composite outcome was analyzed separately in addition to the secondary outcomes of relapse at 90 days and total relapse following completion of initial CDI treatment.

For the composite outcome, patients were considered lost to follow-up if there was no documentation of clinical status in the medical record by 30 days following completion of treatment. The date of last known follow-up was determined by the date of discharge from the hospital or the last known outpatient clinic visit.

### Statistical Analysis

We performed multiple imputation to estimate missing values for laboratory and clinical data using a logistic regression model under the missing-at-random assumption [23–25]. Ten data sets were imputed, and pooled estimates were used for the analyses. Outcome variables used in the main analysis were not imputed.

Patient characteristics by treatment group were presented as counts and percentages for categorical variables and medians and IQRs for continuous variables if they were skewed; we tested for differences using Mann-Whitney or chi-square tests. For the primary composite outcome, time 0 was the date of CDI diagnosis; patients without an event by 30 days of treatment completion were censored, and patients were censored earlier by time of last known follow-up. The primary analysis was a time-to-event analysis based on cause-specific Cox proportional hazards comparing the rate of the composite outcome following treatment with fidaxomicin vs vancomycin and to account for the competing risk of death from other causes. Univariate and multivariate proportional hazards models were evaluated. Candidate variables were included in a preliminary model, including baseline patient characteristics and disease severity markers that were related to the exposure as possible factors confounding the relationship with the outcome. The variables were then removed from the preliminary model in a stepwise approach based on the collapsibility approach (variables were kept in model if there was a change in β coefficient by 20% if eliminated). Type of immunosuppression and severity were forced into the model per clinical judgment; we limited the number of confounders to 4 based on the number of study outcomes [26]. The proportional hazards assumptions were checked via graphical assessment of Schoenfeld residuals and log(-log) plots [27].

The components of the composite outcome, including clinical failure, relapse at 30 days, and CDI-related death, as well as the secondary outcomes of relapse at 90 days and total relapse, were examined individually with a cause-specific Cox proportional hazards model. Unlike the primary analysis of the composite outcome, time 0 was the date of treatment completion for relapse by 30 or 90 days; patients without an outcome were censored at 30 or 90 days or at the date of last known follow-up.

Ten patients in the vancomycin group continued CDI treatment for >30 days, as opposed to none in the fidaxomicin group, creating a potential for immortal time bias when evaluating 30-day relapse in the composite outcome. Immortal time bias occurs when participants have an interval during which the outcome event cannot occur in 1 of 2 treatment arms [28]. Accordingly, we conducted a sensitivity analysis excluding these patients to assess the impact of immortal time bias on the results. In the primary analysis, treatment was based on CDI therapy administered for at least 72 hours. We conducted a sensitivity analysis to evaluate the intention-to-treat effect by assessing the relationship between the first dose of CDI therapy received and the composite outcome. In addition, we conducted a subgroup analysis to evaluate the impact of type of test used to diagnose CDI on the composite outcome.

All statistical analyses were completed with R Studio software version 4.1.2 (R Core Team) or SPSS version 28 (IBM). *P* < .05 was considered statistically significant unless otherwise indicated.

## RESULTS

### Patient Characteristics

**Figure 1.**
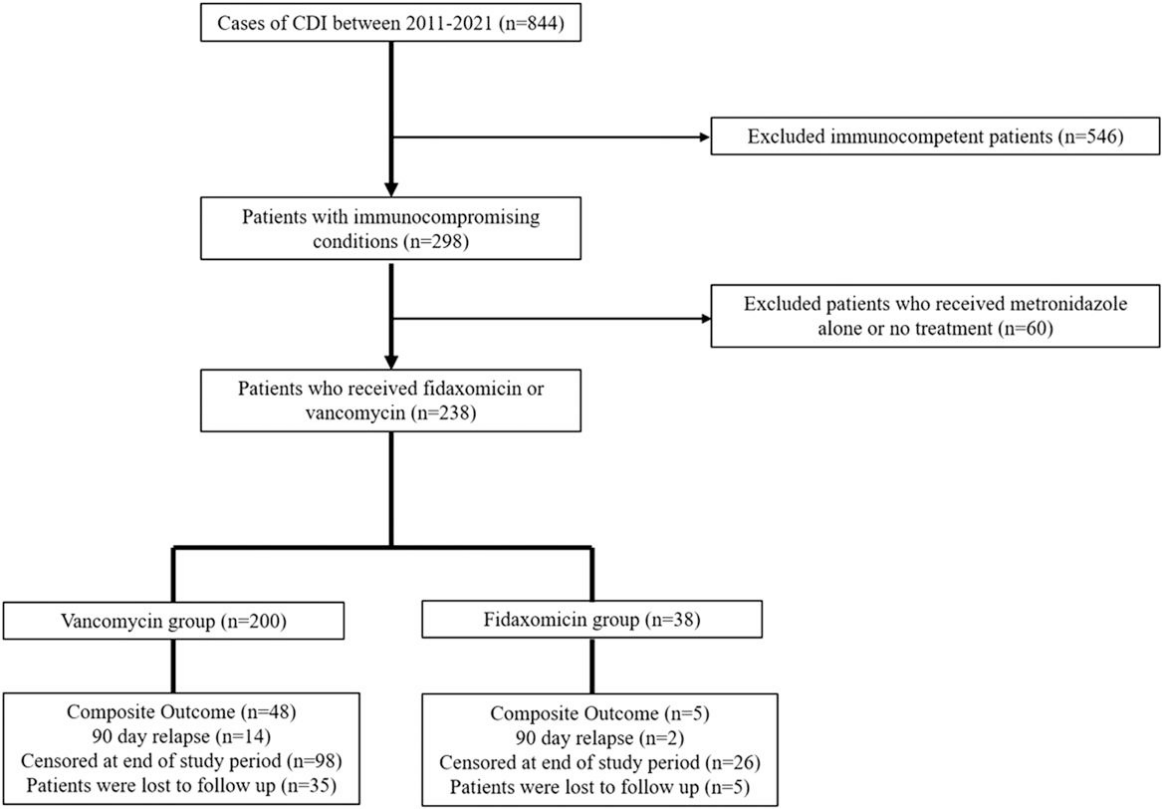
Flowchart of study patient selection. CDI, *Clostridioides difficile* infection.

A total of 844 patients were diagnosed with CDI during the study period: 1 March 2011 to 31 December 2021. Of 298 patients with immunocompromising conditions, 43 were excluded for receiving metronidazole alone for treatment and 17 for not receiving any CDI-directed treatment, resulting in a final sample size of 238 (Figure 1), of whom 38 received fidaxomicin and 200 received vancomycin. There were 68 (28.6%) patients who were treated for >14 days: 11 (28.9%) in the fidaxomicin arm vs 57 (28.4%) in the vancomycin arm. Concurrent antibiotics were being given in 45 (36.6%) patients: 3 (16.7%) in the fidaxomicin group and 42 (40%) in the vancomycin group.

Patients who received fidaxomicin were significantly less likely to be male (26.2% vs 51.5%, *P* < 0.01), to have had community-acquired infection (9.5% vs 31.1%, *P* = .03), and more likely to be undergoing gastric acid suppression (78.9% vs 55.4%, *P* < 0.01), when compared with the vancomycin treatment group (Table 1). There were no other significant differences in demographic and clinical characteristics by treatment. Forty patients were lost to follow-up: 5 in the fidaxomicin group and 35 in the vancomycin group. Patients with loss to follow-up were comparable to those without loss to follow-up except that patients who were censored at the time of last known follow-up were older (66.9 vs 62.7 years, *P* = .04). There were no differences in markers of severity, including intensive care unit stay, location of testing, or toxin positivity.

**Table 1.** Baseline Demographic and Clinical Characteristics of Patients by Treatment After Multiple Imputation

	Fidaxomicin (n = 38)	Vancomycin (n = 200)	*P* Value
Age, y^a^	62.9 (56.7–74.1)	62.5 (53.4–70.7)	.36
Male	10 (26.2)	103 (51.5)	<.01
White	31 (80.5)	148 (74.1)	.42
Hispanic	3 (8.4)	11 (5.3)	.49
Type of immunosuppression			.29
Solid organ transplant	19 (50)	57 (28.5)	
Bone marrow transplant	5 (13.2)	19 (9.5)	
Leukemia/lymphoma	5 (13.2)	43 (21.5)	
Solid tumor	4 (10.5)	40 (20.0)	
Immunomodulator^b^	5 (13.2)	41 (20.5)	
CCI^a^	5 (3,7)	5 (3,8)	.50
Dialysis	7 (18.4)	16 (8.0)	.07
CDI in past 6 mo	2 (5.3)	2 (1.2)	.09
Any history of CDI	7 (18.4)	22 (11.0)	.23
Gastric acid suppression	30 (78.9)	111 (55.4)	<.01
Location prior to diagnosis			.03
Hospital acquired	20 (53.4)	82 (41.1)	
Health care associated	14 (37.1)	56 (27.9)	
Community acquired	4 (9.5)	62 (31.1)	
CDI test type			.45
Toxin and antigen assay	20 (51.3)	116 (58.2)	
*NAAT* (toxin gene)	18 (49.7)	84 (41.8)	
WBC count^a^	6900.0 (4950–12 200)	7600.0 (3900–12 775)	.96
Severe CDI	16 (42.4)	75 (37.7)	.59
ICU stay	8 (19.7)	37 (18.4)	.84
Antecedent antibiotic use	30 (77.9)	146 (73.0)	.55
No. of antecedent antibiotics^a^	2 (1,3)	1 (0,2)	.07
Antibiotics during treatment	18 (47.4)	105 (52.5)	.60
Loss to follow-up	5 (13.2)	35 (17.5)	.64
Time to loss of follow-up^a,c^	23.5 (10.5–35)	15.5 (6–30)	.39

Data are presented as median (IQR) or No. (%). Chi-square was used for all testing unless otherwise specified.

Abbreviations: CCI, Charlson Comorbidity Index; CDI, *Clostridioides difficile* infection; ICU, intensive care unit; WBC, white blood cell count.

^a^Mann-Whitney *U* test.

^b^Immunomodulators: infliximab, azathioprine, tocilizumab, basiliximab, 6-mercaptopurine, cyclophosphamide, cyclosporine, imatinib, high-dose steroid, adalimumab, ixekizumab, vedolizumab, leflunomide, nivolumab, sorafenib, tofacitinib.

^c^Only for patients who were lost to follow-up.

### Clinical Outcomes

A total of 42 (17.6%) patients developed the composite outcome, including 6 (2.5%) who had clinical failure and 26 (11.7%) who relapsed within 30 days of completing treatment. There were 42 patients who relapsed within 90 days; 93% of all relapses were diagnosed by a positive stool test result, and the others were based on clinician judgment to treat recurrent diarrhea. There were 17 deaths in the cohort during the follow-up period, with 10 deaths adjudicated as CDI related while the rest were considered CDI-unrelated deaths. The composite outcome occurred in 4 (10.5%) patients in the fidaxomicin group, as opposed to 38 (19.0%) in the vancomycin group. The distribution of the primary and secondary outcomes by treatment group is displayed in Table 2.

**Table 2.** Primary and Secondary Outcomes by Treatment Group

	Fidaxomicin (n = 38)	Vancomycin (n = 200)	Total (N = 238)
Primary outcome: composite^a^	4 (10.5)	38 (19.0)	42 (17.6)
Secondary outcomes			
Clinical failure	2 (5.3)	4 (2.0)	6 (2.5)
Relapse			
30 d^b^	1 (2.9)	25 (13.4)	26 (11.7)
90 d^c^	2 (5.9)	14 (8.6)	16 (8.2)
Total	3 (8.6)	39 (20.9)	42 (18.9)
Death related to CDI	1 (2.6)	9 (4.6)	10 (4.3)
Other causes of death	5 (13.2)	7 (3.5)	12 (5.0)

Abbreviation: CDI, *Clostridioides difficile* infection.

Data are presented as No. (%).

^a^Composite outcome: clinical failure, relapse at 30 days, and death related to CDI.

^b^After exclusion of patients who died (n = 222).

^c^After exclusion of patients who had clinical failure, died, or had 30-day relapse (n = 196).

Patients who developed the composite outcome were more likely to have been diagnosed with a toxin test and to have received antibiotics when compared with those who did not develop the composite outcome (Table 3)

**Table 3.** Baseline Demographic and Clinical Characteristics of Patients by the Composite Outcome After Multiple Imputation

	Composite Outcome (n = 42)	No Composite Outcome (n = 196)	*P* Value
Age, y^a^	63.6 (55.6–72.4)	62.2 (53.3–70.8)	.18
Male	25 (59.5)	25 (59.5)	.32
White	30 (71.4)	149 (76.0)	.54
Hispanic	2 (4.8)	12 (6.1)	.91
Type of immunosuppression			.58
Solid organ transplant	16 (38.1)	60 (30.6)	
Bone marrow transplant	3 (7.1)	21 (10.7)	
Leukemia/lymphoma	8 (19.0)	40 (20.4)	
Solid tumor	7 (16.7)	37 (18.9)	
Immunomodulator	8 (19.0)	38 (19.4)	
CCI^a^	6 (3.5–8)	5 (3–8)	.44
Dialysis	5 (11.9)	18 (9.2)	.59
CDI in past 6 mo	1 (2.4)	3 (1.5)	.57
Any history of CDI	5 (11.9)	24 (12.2)	.94
Gastric acid suppression	27 (64.3)	114 (58.2)	.46
Location prior to diagnosis			.92
Hospital acquired	17 (40.5)	86 (43.9)	
Health care associated	14 (33.3)	56 (28.6)	
Community acquired	11 (26.2)	55 (28.1)	
CDI test type			.05
Toxin and antigen assay	30 (71.4)	106 (54.1)	
*NAAT* (toxin gene)	12 (28.6)	90 (45.9)	
Severe CDI	18 (42.9)	74 (37.8)	.63
ICU stay	11 (26.2)	33 (16.8)	.17
Antecedent antibiotic use	37 (88.1)	139 (70.9)	.034
No. of antecedent antibiotics^a^	2 (1–3)	1 (0–2)	.011
Antibiotics during treatment	23 (54.8)	100 (51.0)	.66

Data are presented as median (IQR) or No. (%). Chi-square was used for all testing unless otherwise specified.

Abbreviations: CCI, Charlson Comorbidity Index; CDI, *Clostridioides difficile* infection; ICU, intensive care unit.

^a^Mann-Whitney *U* test.

### Multivariable Model

In the multivariate model following adjustment for confounding variables, fidaxomicin was associated with a 72% reduction in the hazard of developing the composite outcome as compared with vancomycin (hazard ratio [HR], 0.28; 95% CI, .08–.93; Table 4). After adjustment, the relationship between fidaxomicin and other causes of death was not statistically significant (HR, 3.3; 95% CI, .90–11.70)

**Table 4.** Unadjusted and Adjusted Cause-Specific Proportional Hazard Model for Fidaxomicin vs Vancomycin

	Hazard Ratio (95% CI)	
	Unadjusted Model	Adjusted Model^a^
Composite outcome^b^: fidaxomicin	0.54 (.19–1.51)	0.28 (.08–.93)
Secondary outcomes		
Clinical failure	2.56 (.47–14.0)	0.89 (.09–8.67)
Relapse		
30 d	0.21 (.03–1.56)	0.15 (.02–1.15)
90 d	0.65 (.15–2.87)	0.50 (.11–2.35)
Total	0.36 (.11–1.18)	0.27 (.08–.91)
CDI-related death	0.56 (.07–4.45)	0.31 (.04–2.67)

Other causes of death: adjusted hazard ratio for composite outcome, 3.3 (95% CI, 0.90–11.70).

Abbreviation: CDI, *Clostridioides difficile* infection.

^a^Adjusted for sex, number of antecedent antibiotics, CDI severity, and type of immunosuppression.

^b^Composite outcome: clinical failure, relapse at 30 days, and death related to CDI.

An exploration of the relationship between treatment and the individual components of the composite outcome and secondary outcomes is shown in Table 4. The hazard of total relapse was significantly lower in the fidaxomicin group vs the vancomycin group in the multivariable adjusted model (odds ratio, 0.27; 95% CI, .08–.91).

A subgroup analysis evaluating the type of test used to diagnose CDI was conducted to see if test type had any impact on clinical efficacy. Fidaxomicin significantly reduced the hazard of the composite outcome in the toxin-positive group (HR, 0.11; 95% CI, .01–.86) but not in the NAAT positive group (HR, 0.70; 95% CI, .14–4.50) after adjusting for sex, number of antecedent antibiotics, CDI severity, and type of immunosuppression.

We conducted 2 sensitivity analyses to test our assumptions. In the sensitivity analysis that excluded 10 patients in the vancomycin group who received treatment for >30 days (median, 44 days; IQR, 37–61), the hazard of the composite outcome was 0.26 with fidaxomicin (95% CI, .08–.87) as compared with vancomycin, which was similar to the primary analysis.

In a sensitivity analysis based on intention to treat (n = 7 with vancomycin as first dose, n = 9 with fidaxomicin as first dose), fidaxomicin was still associated with a 74% reduction in the hazard of the composite outcome when compared with vancomycin after adjustment (HR, 0.26; 95% CI, .08–.92).

## DISCUSSION

We evaluated the clinical effectiveness of fidaxomicin vs vancomycin in preventing CDI treatment failure in patients with immunocompromising conditions. Our findings suggest that fidaxomicin offers superior protection against CDI treatment failure when compared with vancomycin in this population.

Since this retrospective study was conducted from 2011 to 2021, vancomycin was the agent largely prescribed for initial and recurrent episodes of CDI, consistent with the IDSA CDI guideline recommendations at the time when the study was conducted [19]. In 2021, the guidelines of the IDSA and European Society of Clinical Microbiology and Infectious Diseases were updated to recommend fidaxomicin as the first line of treatment [12, 29]. Although our institutional guidelines during the time frame studied recommended fidaxomicin to be used in a larger proportion of patients than that seen in our study, factors such as limited insurance coverage and cost may have played a role in fidaxomicin being used less frequently.

Notably, in our study, patients who received fidaxomicin had a 72% reduction in the hazard of treatment failure. These results are similar to a post hoc analysis of oncology patients who participated in randomized controlled trials for treatment of CDI in which the odds of recurrence in the fidaxomicin group was significantly lower (odds ratio, 0.37) than the vancomycin group [18]. Yet, a retrospective study evaluating the efficacy of fidaxomicin vs vancomycin in allogenic stem cell transplants did not find a difference in clinical cure [18]. There were several methodologic differences between that study and ours. One, in that study, vancomycin was given for a prolonged period, at a mean 21 days vs only 9 days for fidaxomicin treatment. So, its results were skewed to favor vancomycin. Furthermore, CDI testing for the majority of that study was conducted with an NAAT. In addition, many of its patients had acute graft-vs-host disease; thus, if stool was sent for *C difficile* testing, patients may have been misclassified as having *C difficile*–associated disease. Finally, that study was limited to recipients of stem cell transplant, whereas our study included a variety of hosts who were immunocompromised (BMT, SOT, and immunomodulator treated).

The total relapse rate (30 and 90 days) was significantly lower in the fidaxomicin group vs the vancomycin group, although the overall rate of relapse reported in our study was 19.9%, which is slightly lower than previously reported relapse rates of 25% to 40% in other studies of patients who were immunocompromised [2, 30, 31].

Interestingly, a subgroup analysis based on test type showed benefit of using fidaxomicin only in toxin-positive CDI cases, not NAAT positive. Fewer patients developed the composite outcome in the NAAT group. Another possible explanation is the presence of a group of patients who were colonized with *C difficile* but did not have true infection, so treatment did not affect the clinical outcome.

Although there was a signal toward an increased hazard of other causes of death in the fidaxomicin group, we investigated all causes of death and found that these patients died from chronic conditions that were present prior to CDI diagnosis; in fact, many were discharged to hospice or had care withdrawn due to progression of their underlying disease.

There are several strengths to this study. The study had a larger cohort of patients who were immunocompromised than has previously been studied and represents “real-world use” of fidaxomicin and vancomycin in this population. The use of our statistical models allowed us to control for potential confounders, given the retrospective nature of the study design. In addition, we accounted for the competing risk of death from other causes. We also assessed for immortal time bias based on some patients getting long courses of vancomycin.

There are several potential limitations in this study. This was a retrospective single-center study with a limited sample size. There were some baseline variables missing and some patients who were lost to follow-up. Multiple imputation was used for missing baseline variables, and the use of a Cox proportional hazards model allowed us to censor patients at the time of last known follow-up. From a diagnostic testing perspective, we did include patients who had only a positive NAAT result. Although NAAT is licensed for use in diagnosis, patients with only nucleic acid test positivity may reflect colonization rather than true disease. But given that these patients have historically met the case definition of CDI and that this test was being used at the time of the study, we included these patients. Another potential source of bias was that all treatment decisions were determined by the treating physician, which may have led to confounding by indication. Although we adjusted for potential confounders, it is possible that residual confounding remains. We categorized patients by treatment group based on the agent used for ≥72 hours, but a small number of patients received a different agent for the first dose. A sensitivity analysis exploring an intention-to-treat approach found results similar to the primary analysis. Also, treatment duration varied slightly in the vancomycin group vs the fidaxomicin group, which created the potential for immortal time bias for the relapse outcomes. We performed a sensitivity analysis excluding patients treated with vancomycin for >30 days and demonstrated results similar to the primary analysis.

In conclusion, our analysis confirms that fidaxomicin should be first line for treating CDI in patients with immunocompromising conditions and that such use was associated with a reduced risk of treatment failure as reflected in our composite outcomes. Future multicenter studies are needed to confirm our findings.

## Notes

***Acknowledgments.*** We acknowledge Caitlin Trottier, MD, who helped adjudicate the causes of death in this cohort.

***Patient consent statement.*** The Tufts Medical Center Institutional Review Board approved this study. The board approved a waiver of consent since the study does not include factors necessitating patient consent.

***Financial support*.** This work was supported by a grant from Merck & Co; the Francis P. Tally, MD, fellowship in infectious disease at Tufts Medical Center; the Tupper Research Fund; and the National Center for Advancing Translational Sciences, National Institutes of Health (UL1TR002544 to M. A.).
